# Achieving Superlubricity: Development of Multilayer Co-Doped DLC Coatings and Tribological Evaluation with Eco-Friendly Base Oil and Low-SAPS Oil Formulations

**DOI:** 10.3390/ma18040847

**Published:** 2025-02-14

**Authors:** Mobeen Haneef, Manuel Evaristo, Liuquan Yang, Ardian Morina, Bruno Trindade

**Affiliations:** 1 ARISE—Advanced Production and Intelligent Systems, Department of Mechanical Engineering, University of Coimbra, Rua Luis Reis Dos Santos, 3030-788 Coimbra, Portugal; manuel.evaristo@dem.uc.pt; 2 CEMMPRE—Centre for Mechanical Engineering Materials and Processes, Department of Mechanical Engineering, University of Coimbra, Rua Luis Reis Dos Santos, 3030-788 Coimbra, Portugal; 3 Institute of Functional Surfaces (iFS), School of Mechanical Engineering, University of Leeds, Leeds LS2 9JT, UK; l.q.yang@leeds.ac.uk (L.Y.); a.morina@leeds.ac.uk (A.M.)

**Keywords:** cobalt, doped-DLC, cobalt-doped DLC, multilayer coatings, mechanical properties, adhesion, tribology, ashless, sulphur free, superlubricity

## Abstract

To address modern tribological challenges—reducing friction and wear to conserve resources while minimising environmental impact—cobalt-doped DLC (Co-DLC) coatings were developed. These nanometric multilayer coatings, designed to retain key properties such as hardness, reduced modulus, and substrate adhesion, were fabricated using non-reactive DC magnetron sputtering (DCMS). The multilayer structure was achieved by controlling the planetary substrate holder’s rotational speed. Characterisation of microscopic, chemical, structural, and mechanical properties was performed using techniques including FEI-SEM, EDS, XRD, TEM, Raman spectroscopy, scratch adhesion testing, and nanoindentation. Tribological performance was evaluated under boundary and fully flooded lubrication using PAO4 base oil and formulations with ashless, sulphur-free AW and EP additives. The coatings exhibited a granular surface morphology, columnar cross-sections, and amorphous structure. Increased dopant concentrations slightly enhanced graphitisation and significantly improved adhesion, though hardness and reduced modulus decreased. Tribological testing revealed superlubricity in several coating–oil combinations and significantly reduced wear rates with higher dopant levels and new additives. A phosphate ester additive without an amine group achieved the lowest COF values, while one with an amine group yielded minimal wear rates. These findings highlight the potential of Co-DLC coatings and tailored additives to minimise friction and wear effectively.

## 1. Introduction

Diamond-like carbon (DLC) coatings are amorphous carbon films that possess a unique combination of properties from both diamond and graphite. The inclusion of both carbon phases in a single coating endows them with high hardness, abrasion resistance, self-lubricity, biocompatibility, thermal stability, and oxidation resistance [1]. These properties make DLC coatings highly desirable for various applications nowadays, although this has not always been the case. First developed by Schmellenmeier in 1953 [2], DLC coatings were initially limited by high residual stresses, low toughness, reduced capability of elastic recovery, and occasionally problematic inertness when chemical responsiveness was required. These drawbacks were significant obstacles to the deployment of such coatings for applied mechanical and tribological applications. However, recognising the intrinsic beneficial properties of these coatings, efforts to mitigate these problems began in the mid-1980s [3]. These efforts focused on two main strategies: developing different deposition methods for these coatings and doping DLC with foreign elements (also known as nanocomposites of DLC) to enhance their properties.

To date, many different deposition methods have been developed for the production of DLC coatings, typically based on physical vapour deposition (PVD), chemical vapour deposition (CVD), and hybrid approaches. Among these techniques, non-reactive direct current magnetron sputtering (DCMS), a PVD method, has emerged prominently due to several benefits [4,5]. These benefits included a simple setup, the use of uncomplicated targets of the materials to be deposited, a noble gas (usually argon) to generate and sustain plasma for sputtering to take place, an electric power supply(s), and a room to keep the equipment. The scalability from laboratory to industrial scale has also contributed to the success of this method. DLC coatings deposited by this method exhibit optimal mechanical and tribological properties [6,7,8,9,10,11]. However, there remains room for improvement, particularly for DLC coatings tailored for specific mechanical and tribological applications. These coatings are potential candidates for achieving superlubricity in the tribological contacts where the coefficient of friction (COF) drops below 0.01 [12] to address the problem of 20% energy loss every year due to friction, which contributes to pollution and global warming [13]. Previously, the authors reported a methodology for depositing multilayer Ti-doped DLC coatings to enhance mechanical properties [14]. When these Ti-DLC coatings were tested with a PAO4 base oil formulation and an ashless, sulphur-free phosphate ester, superlubricity was achieved, along with reduced wear rates [12].

DLC coatings are often doped with transition metals that form short- or long-range order carbides when they react with the DLC matrix. These doping elements include Ti, W, V, Cr, and Fe. When doped into DLC coatings, these elements help to reduce internal stresses, improve adhesion, and enhance tribological performance [15,16,17]. Cobalt (Co), on the other hand, has a lower tendency to form carbides, even when doped in a carbon-based material by PVD methods [18]. The known carbides of Co are made by wet chemical routes, usually for catalytic processes for petrochemical industries [19,20]. This gives an opportunity to study the DLC coatings doped with Co and without the formation of the carbide phase in the coatings. Co, at the same time, possesses several attractive properties. These properties include a high melting point (1495 °C) [21], high strength at room and elevated temperatures, along with optimal corrosion and sulfidation resistance [22]. Most importantly, Co has proven its capability in mechanical and tribological applications by showing high wear resistance in bulk [23] and in coatings [24]. Co is a reactive element that forms tribofilms by reacting with the additives in lubricating oils [25]. Tribofilms typically form with a thickness of a few tens of nanometers, serving to reduce wear, friction, and oxidation [26]. However, reports related to Co-doped DLC coatings could not be found. These attributes make cobalt an appealing dopant for DLC coatings, and the emerged mechanical and tribological properties are worth exploring. To date, Co-DLC coatings have been reported to have applications in the potent applied areas of ultrahigh density recording media [27], in spintronics devices [28], and in magnetic sensor applications [29].

The literature on Co-doped DLC (Co-DLC) coatings is scarce for mechanical and tribological applications. Y. Tang et al. [18] reported the development of Co/DLC coatings by biassed target ion beam deposition (BTIBD) with a Co content of 24 at.%, concluding that Co does not interact significantly with carbon in the coatings and does not greatly alter the microstructure. B. Li et al. [30] studied Co-DLC (10–36 at.% Co) coatings for biosensor applications and similarly found that Co does not interact with carbon in the DLC, although they did not report the mechanical properties of the coatings. L. Ma et al. [31] also reported on the sensor and field emission applications of Co-DLC coatings deposited by RF sputtering, concluding that Co doping does not significantly affect the sp^2^/sp^3^ ratios in the DLC coatings nor form a carbide phase. X. Ling et al. [32] studied Fe- and Co-doped DLC coatings for field emission applications via electrochemical deposition (ECD) and concluded that Co incorporation does not affect the bonding in DLC coatings and can exist in small metallic domains ranging from 4 to 8 nm. Although Co-DLC coatings have been developed as thin films, their mechanical properties, such as hardness and adhesion to substrates, have not been thoroughly investigated. Additionally, while DLC coatings are well-documented for mechanical and tribological applications, these properties of Co-DLC coatings have yet to be explored. The work conducted can provide insights into the mechanical and tribological response of Co-doped DLC coatings, as well as it would help other researchers working on the magnetic properties and a detailed overview of the mechanical properties of these coatings for applied applications.

In addition to enhancing mechanical properties, DLC coatings are often paired with lubricating oils to further reduce wear and friction, with the potential to achieve superlubricity. Traditionally used lubricating oil additives contain environmentally harmful substances. Therefore, there is a growing need for oils and additives that are low in or free of sulphonated ash, phosphorus, and sulphur (SAPS), in order to maintain comparable performance to conventional additives while being safer for the environment.

In this work, three Co-doped DLC coatings, with Co contents of 4, 7, and 9 at.%, were developed in a nanometric multilayer formation of consecutive Co-DLC and DLC layers (Co-DLC/DLC). These coatings were deposited with a multilayer microstructure to achieve better mechanical properties [12,14] using unbalanced DCMS.

The developed coatings were first studied for their morphological, structural, and mechanical properties. Subsequently, the tribological performance of these coatings was evaluated under fully flooded oil conditions using a synthetic base oil and two antiwear (AW) and extreme pressure (EP) additives, which were free from sulphur and sulphonated ash. The results are presented, compared, and discussed.

## 2. Materials and Methods

Co-doped DLC (Co-DLC) coatings, with three different concentrations of the dopant, and one undoped DLC coating as a reference for comparison of the properties, were deposited on a silicon (Si) substrate (p-type, monocrystalline, (100) ± 0.5°, 15 mm wide, 15 mm long, 0.525 mm thick, from Siegert Wafer, Aachen, Germany) and on quenched and tempered steel (AISI M2, hardness 63 HRC, 50 mm diameter, 3.0 mm thick, from Thyssenkrupp Materials, Iberica, Spain). The coatings deposited on Si substrates were used for morphological, structural, and chemical analysis. In contrast, the coatings deposited on steel substrates were used for tribological evaluation, nanoindentation hardness testing, and scratch adhesion tests.

The preparation of the substrates and the deposition conditions were the same as those used in previous work [14]. The deposition of the coatings was performed using a TEER Coatings Limited (Droitwich Spa, UK) semi-industrial magnetron sputtering system (UDP 650/4) with one 99.95% pure chromium (Cr) target, two 99.99% pure graphite (C) targets, and one 99.99% pure C target embedded with fourteen 99.90% pure Co pellets. The C targets measured 380 mm in length and 175 mm in width, while the Co pellets had a diameter of 20 mm and a thickness of 5 mm.

**Figure 1:**
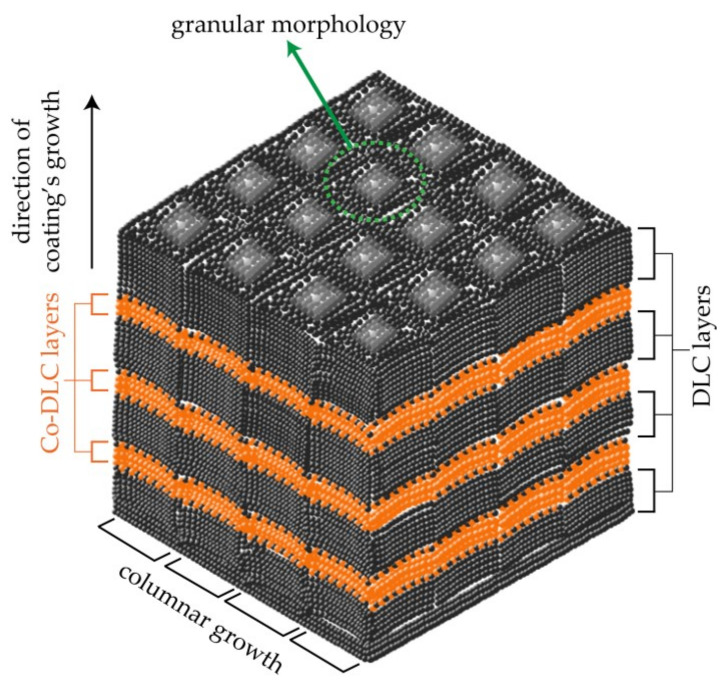
Schematic of the multilayer structure of Co-doped DLC (Co-DLC/DLC = MCo-DLC) coatings.

Prior to the deposition of all coatings, a ‘C + Cr’ gradient adhesive interlayer with an average thickness of 0.33 µm was deposited on the substrates to enhance adhesion. This interlayer was deposited at a rotational speed of 12 rpm for the substrate holder across all coatings, including one undoped DLC and three multilayer Co-doped DLC coatings. The three Co-doped DLC coatings were deposited at 1 rpm. This slower rotation facilitated the formation of a nanometric multilayer microstructure consisting of alternating layers of Co-DLC and DLC, by using two kinds of targets: ‘graphite’ and ‘graphite + Co pellets’. Contrarily, the undoped DLC coating was deposited at 12 rpm since no distinct materials were present to form a multilayer microstructure. The microstructure of the multilayer coatings is presented schematically in Figure 1; the microstructure of the interlayer is not included in this figure.

The coating deposition conditions and the names of the coatings to be used in this research work are presented in Table 1, these parameters are for coating’s deposition and not for the interlayer. Three multilayer Co-doped DLC coatings with different atomic concentrations of Co dopant were deposited, referred to as *M*Co-DLC1, *M*Co-DLC2, and *M*Co-DLC3. One pure DLC coating, referred to as DLC, was also deposited as a reference for comparison of the properties of interest.

To achieve three different concentrations of Co in the DLC coatings, three power rating values were used for the ‘C + Co’ target: 160 W, 305 W, and 450 W. Two C targets were used due to the lower sputtering rate of C. For the Co-doped coatings, a longer deposition time was chosen for coatings with low Co content and vice versa to account for the higher deposition rate of Co compared to C and to maintain nearly identical coating thicknesses for comparison of the properties. During the deposition of the interlayer and all coatings, a negative 110 V bias was applied to the substrate to consolidate the coatings, and the power at the two C targets was kept constant at 1750 W.

A field emission—scanning electron microscope (FEI-SEM—Hitachi Merlin Gemini III, Krefeld, Germany) was used for the morphological analysis of coatings concerning cross-sections and surfaces. It was also used to measure the thickness of the interlayer and coatings. To observe the cross-section, a coated Si substrate was cleaved using a diamond tip cutter to obtain a freshly prepared cross-section. For surface morphological analysis, the Si substrate with deposited coatings, kept in dust-proof packaging, was used after cleaning them with an air blower before analysis. The SEM micrographs were also used to calculate the surface granular sizes of the coatings, which emerged from the cauliflower-like surface morphology, by processing the micrographs using ImageJ software (version 1.54g; Java 1.8.0_345 64-bit).

EDS—Bruker Nano GmbH (Berlin, Germany)—XFlash Detector 610M was employed to analyse the elemental composition of the coatings. The EDS measurements were performed using an EDS sensor available with a different SEM (Hitachi SU3800, Tokyo, Japan) due to the better calibration of the EDS equipment. This analysis was performed at 10 kV of accelerating voltage of the electron beam for all the samples.

XRD—Panalytical X’pert (Philips XPert, Malvern Panalytical Ltd., Malvern, UK) analysis was performed on the coatings deposited on the Si substrate. The apparatus was equipped with a Co X-ray tube (generating voltage 40 kV and tube current 35 mA) for which the X-rays had a wavelength of kα = 1.78897 Å. It was used for structural analysis of the coating in Bragg–Brentano geometry, covering a 2θ° range from 30° to 60° with a step size of 0.025°. The analysis was also performed for the coatings deposited on M2 steel samples as well as in grazing incidence mode with an incident angle of 2° to avoid interference from the ‘C + Cr’ gradient interlayer and the substrate.

FEI-TEM—Titan Themis Cubed 300 (Hillsboro, OR, USA) was used to investigate the internal structure of the Co-DLC coatings. The EDS tool in the TEM was employed to explore the distribution and concentration of the Co dopant in the multilayer coatings. Another TEM (TESCAN TENSOR 4D-STEM, Brno-Kohoutovice, Czech Republic) was used to obtain electron diffraction patterns (EDPs) of the coatings. The EDPs were obtained at several points using diffraction imaging with a hybrid-pixel direct electron diffraction detector. For the analysis of the coatings by both TEM instruments, a focused ion beam (FIB—ThermoFisher, FEI Helios G4 CX DualBeam, Hillsboro, OR, USA) was used to prepare cross-sectional samples consisting of a thin lamella, which were supported on a sample holder.

Raman spectroscopic analysis (Renishaw—inVia Raman Microscope, Wetzlar, Germany) was performed on the undoped DLC and Co-DLC coatings to estimate the extent of graphitisation triggered by the dopant in the coatings. The analysis was conducted at room temperature using a laser with a 532 nm wavelength, 5% intensity of laser beam, a 100× magnification microscope objective, and an exposure time of 15 s for each of the three spots per sample.

The hardness (H) and reduced modulus (E*) of the coatings were measured using nanoindentation (Micro Materials Ltd., Wrexham, UK, NanoTest) equipped with a Berkovich indenter. This analysis was performed on coatings deposited on both Si substrate and AISI M2 steel. The equipment was operated in load control mode using a 5 mN normal load. Sixteen indents in the form of a 4 × 4 matrix were made on each sample, and average values are reported in Section 3. The elastic–plastic response of the coatings was estimated by calculating the H/E* and H^3^/E*^2^ values [33]. The values of W_e_ were calculated to measure the coating’s toughness and its resistance to cracking [34,35], where W_e_ and toughness are directly proportional to each other. These W_e_ values were calculated using the procedure detailed in [14,36].

A stylus profilometer (Mitotoyo, Surftest SJ-500, Kanagawa, Japan) was used to record the radius of curvature of uncoated and coated samples at six different locations (three scans at 0° and three scans at 90° of relative position) to measure the residual stresses in the coatings. The stresses were calculated using the Stoney equation and according to the methodology given in [37]. The Stoney equation is represented as:

(1) $$\sigma =\frac{E_{s}}{6\left(1-V_{s}\right)}\frac{{hs}^{2}}{hf}\left(\frac{1}{R}-\frac{1}{R_{0}}\right)$$

where E_s_, V_s_, and hs are the Young’s modulus, the Poisson’s ratio and the thickness of the substrate, respectively, hf is the thickness of the coating, and R_0_ is the value of the radius of curvature of the substrate before deposition, and R is the value of the radius of curvature of coating deposited on the same substrate for which R_0_ was measured.

The scratch test (CSEM—Revetest) was performed to quantify the practical adhesion of the coatings deposited on the polished AISI M2 steel. The test was conducted using a Rockwell-C indenter, Moessner, Pforzheim, Germany according to the work of Gonczy et al. [38] using the standard ASTM C1624-22 [39]. Five scratch passes were made on each sample with a force ranging from 0 to 50 N over a length of 5 mm, corresponding to a loading rate of 10 N/mm. The Lc2 adhesive failure values, which correspond to the first delamination of the coating, were determined by careful observation of delamination in the micrographs obtained by an optical microscope. A travelling optical microscope (Leica LH 111 LED, Wetzlar, Germany) was used to identify the location of the first delamination and for capturing the micrographs of the scratches over their complete lengths and at specific spots of the first delamination.

A synthetic base oil, PAO4—isoparaffinic polyalphaolefin from Synfluid—Chevron Phillips Chemicals (The Woodlands, TX, USA), was used for tribological tests, both in pure form and to make oil formulations with oil additives. It had a kinematic viscosity of approximately 17 cSt at 40 °C and approximately 4 cSt at 100 °C (100 cSt = 1 cm^2^/s), a density of 0.819 g/cm^3^, and a CAS number of 68037-01-4.

Two organic oil additives were used to make oil formulations for the tribological tests, both of which were AW and EP in nature. These additives included Duraphos^®^ 178 (alkyl amine isooctyl phosphate) and Duraphos^®^ OAP (octyl dihydrogen phosphate). Duraphos^®^ 178 has the chemical formula C_19_H_42_NPO_4_—C_29_H_63_NPO_4_, and it is a nitrogen-containing (as an amine group) phosphate ester, comprising 5.95–8.17 wt.% phosphorus and 2.69–3.70 wt.% nitrogen, with CAS number 68187-67-7 [40]. Duraphos^®^ OAP has the chemical formula C_8_H_19_PO_4_; it is also a phosphate ester but lacks nitrogen (no amine group), containing approximately 14.73 wt.% phosphorus, with CAS numbers 39407-03-9 and 3991-73-9 [41]. These additives were ashless and sulphur-free.

Both the additives used to make the oil formulations for the tribological tests were from Solvay S.A (Brussels, Belgium). The base oil and additives were provided by TOTAL Energies, Lyon, France, for this work. To evaluate the tribological response of the developed coatings, pure base oil and base oil formulations with three additive amounts (0.1, 0.2, and 0.3 wt.%) of each additive were prepared. The tribological tests were performed under flooded conditions on uncoated and polished AISI M2 steel, DLC, and *M*Co-DLC coatings.

The compositions of the oil formulations are referred to in this work as BO-X-Y, where X is amount of additive and Y is the type of additive in the base oil, e.g., BO-0.1-OAP means PAO4 oil formulation with 0.1 wt.% of Duraphos^®^ OAP but the unformulated base oil is simply referred to as BO.

Atomic Force Microscopy (AFM—Bruker Veeco DiInnova, NY, USA), equipped with a silicon cantilever tip with a tip radius of 6 nm, and White Light Interferometry (WLI—Bruker NPFLEX NPF-12-216, MA, USA) were used to measure the roughness of the coatings. The WLI was equipped with Bruker Vision 64 Map^TM^, version 5.41 Update 3, an image processing software capable of measuring the wear directly on the samples.

**Figure 2:**
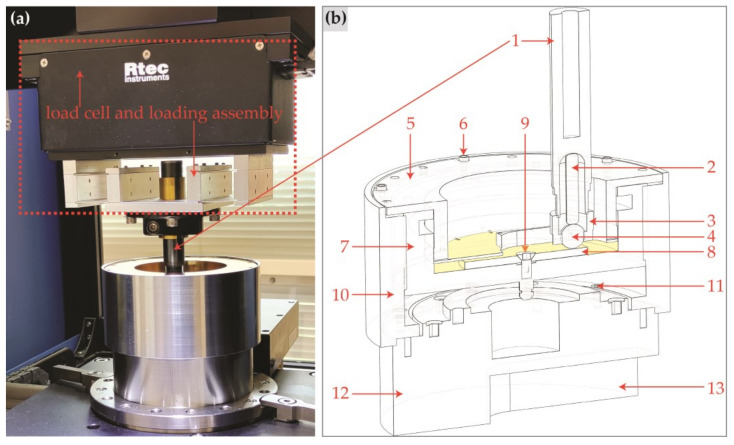
Tribological testing apparatus, (**a**) actual image of the testing apparatus showing load cell and loading assembly, (**b**) a 3D according to scale cross-section of testing apparatus, 1 = shank of ball holding fixture, 2 = ball guide pin, 3 = locking nut for ball, 4 = counterbody ball, 5 = non-rotating and oil recirculating disc, 6 = bolts to tighten the lid, 7 = inner rotating and disc holding enclosure, 8 = sample discs, 9 = sample disc locking bolt, 10 = outer non-rotating enclosure, 11 = bolts to tighten non-rotating outer enclosure, 12 = connection to disc rotating motor via timing belt, and 13 = space for timing belt.

A tribometer (RTEC—MFT 5000, Yverdon-les-Bains, Switzerland) was used to conduct tribological tests in a temperature-controlled laboratory. The temperature during the tests was maintained at 25 ± 2 °C to ensure consistency and minimise the influence of thermal variations on the tribological performance of the coatings. The tests were performed in a unidirectional ball-on-disc mode under boundary lubrication conditions using a 10 mm diameter AISI 52100 steel ball. The steel balls were procured from Luis Aparicio Precision Balls, Barcelona, Spain. A 3D optical profilometer (Bruker—Alicona G4 InfiniteFocus, Leicestershire, UK) was used to measure the roughness of the steel balls; they had an average roughness of 0.04 ± 0.01 µm. The tribological test parameters were as follows: a normal load of 10 N, a Hertzian contact pressure of 1 GPa, a linear speed of 0.55 m/s, and a test duration of 30 min. These conditions translate to approximately 1 km of sliding distance. The contact between the ball and disc was immersed in oil throughout the test. The total volume of base oil and additive was approximately 41 mL for each test. All the oil formulations were freshly prepared before conducting the tribological tests so that the effects of storing the oils can be minimised. The main parts of tribological testing equipment are shown in Figure 2.

The COF values were directly measured using computer-aided software (RTEC MFT 18 R4C, version 18.4.3) integrated with the tribological testing equipment, which calculated the values based on the tangential forces (F_x_) and normal forces (F_z_) between the disc and ball (COF = F_x_/F_z_). After the completion of the tribological tests, the discs and balls were rinsed with n-hexane to remove oil in order to prepare them for further analysis.

The wear volume on the discs resulting from the rubbing of the counterbody ball during the tribological tests was measured using WLI. The wear volume of the balls was calculated by measuring the wear scar diameter using an optical microscope (Leica LH 111 LED). The specific wear rates (SWRs) of the wear tracks on discs and wear scars on the balls were measured according to the methodology defined in [12].

To assess the morphology of the post-tribological tested surfaces of discs and balls, SEM was used. To quantify the chemical composition of the tested surfaces and of any possible tribofilm or transfer layer, EDS was employed.

The software used for data analysis, visualisation, plotting, processing, graphing, and image generation included the following: RTEC Viewer16 (version 1.0.4.0) from RTEC Instruments (Frankfurt, Germany), Solidworks^®^ 2023 (student, version 31.3.0.44) by Dassault Systèmes (Vélizy-Villacoublay, France), Microsoft PowerPoint 365 (version 16.0.18429.20132), Microsoft Excel 365 (version 16.0.18429.20132), OriginLab^®^ OriginPro^®^ 2024 (research, version 10.10.178), Bruker Vision 64 Map^TM^ (version 5.41 Update 3), Gwyddion (open source, version 2.67), and ImageJ (open source, version 1.54g; Java 1.8.0_345 64-bit).

## 3. Results and Discussion

### 3.1. Morphological, Chemical, and Structural Analysis

**Figure 3:**
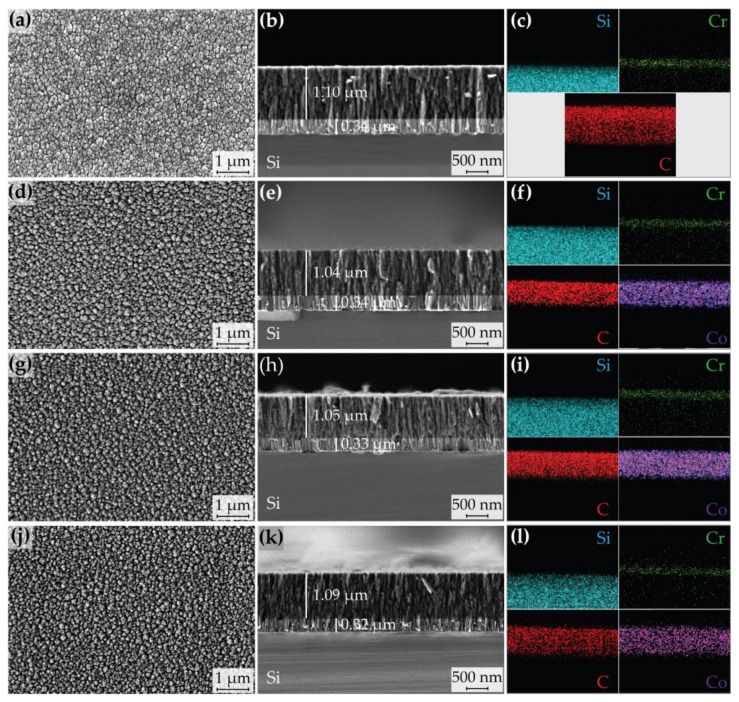
The SEM micrographs of the coating’s surface (**left**), cross-section (**middle**), and EDS elemental maps (**right**) are displayed as follows: (**a**–**c**) DLC, (**d**–**f**) *M*Co-DLC1, (**g**–**i**) *M*Co-DLC2, and (**j**–**l**) *M*Co-DLC3.

The SEM micrographs of DLC, *M*Co-DLC1, *M*Co-DLC2, and *M*Co-DLC3 coatings are shown in Figure 3.

The ‘C + Cr’ gradient adhesive interlayer exhibited a thickness ranging from 0.32 to 0.34 µm and displayed a columnar cross-sectional morphology, consistent with findings in the literature as demonstrated in [42]. The DLC and *M*Co-DLC coatings had thicknesses between 1.04 and 1.10 µm, with an average thickness of 1.07 µm. All the coatings showed a columnar cross-sectional morphology. It is visually apparent that the columns became more refined as the amount of Co dopant increased (Figure 3b,e,h,k).

The surface of all the coatings displayed a gritty, cauliflower-like morphology, originating from the atomic shadowing effect [43], as well as the termination of columns from individual growing fronts Figure 3. The size of these individual granules depends on the structure and size of columns in the coating, which are influenced by the substrate temperature during deposition, the bias applied to the substrates, the working pressure of the gas, and the deposition rate [44].

**Figure 4:**
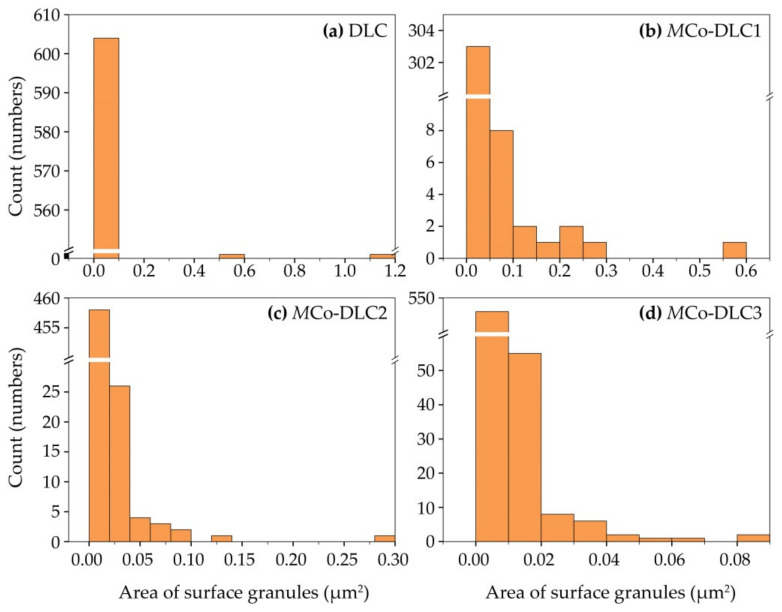
The histograms of granular size distribution for, (**a**) DLC, (**b**) *M*Co-DLC1, (**c**) *M*Co-DLC2, and (**d**) *M*Co-DLC3.

The average calculated granular sizes for the coatings were 0.005, 0.01, 0.005, and 0.003 µm^2^ for DLC, *M*Co-DLC1, *M*Co-DLC2, and *M*Co-DLC3, respectively. The histograms of these granular sizes are presented in Figure 4.

It can be observed from Figure 4 that the DLC coating exhibited the highest degree of monodispersity in surface granular size. In contrast, the *M*Co-DLC coatings displayed a greater degree of sized polydispersity in granular sizes.

**Figure 5:**
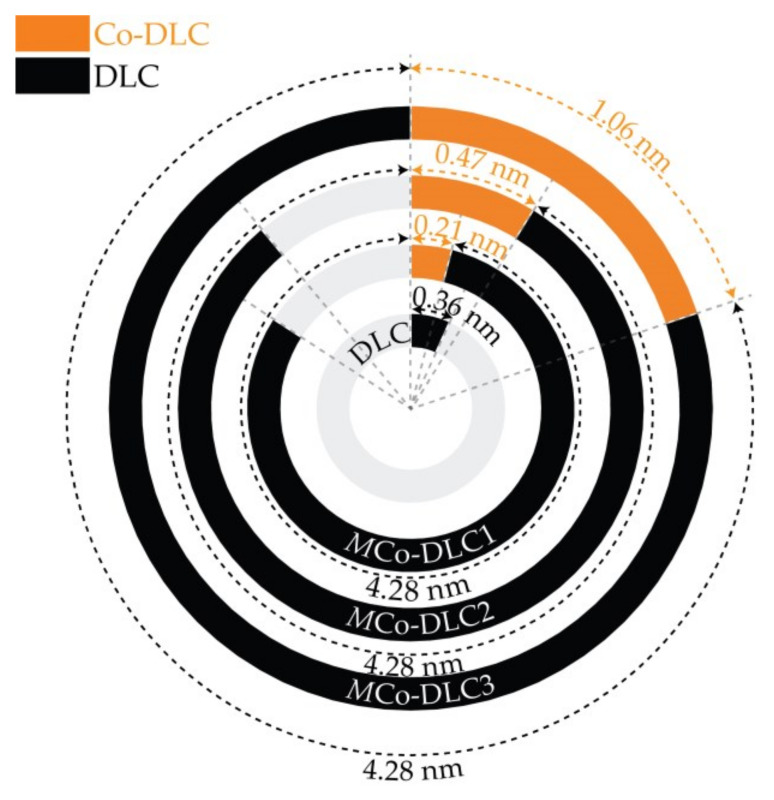
Calculated thickness of DLC and Co-DLC layers per revolution for DLC and the multilayer *M*Co-DLC 1, 2, 3 coatings. The grey shaded area is a background colour and not part of data.

The contribution of different targets toward the thickness of deposited coatings was calculated based on the sputtering rates of the targets, as shown in Figure 5. The undoped DLC coating was deposited over a total duration of 258 min (15,480 s), corresponding to a deposition rate of 4.3 nm/min (0.36 nm/revolution). Two C targets were used to deposit C in the form of DLC; each was powered at 1750 W. This resulted in each C target contributing approximately 0.18 nm of DLC layer per revolution. From the measured and calculated deposition parameters of the undoped DLC coating, it was possible to determine the period between the DLC and Co-DLC layers in the multilayer *M*Co-DLC coatings. These values are shown in Figure 5. The thickness of the Co-DLC layers increased with higher power at the ‘C + Co’ target, resulting from increased sputtering rate at higher power ratings.

The elemental composition measured using EDS for all the coatings is shown in Table 2. It can be observed that Co was incorporated into the coatings with three different amounts: 4.1, 6.9, and 8.7 at.%.

In addition to the coatings shown in Table 2, one more Co-doped DLC coating was deposited to estimate the maximum amount of Co in the layers of Co-doped multilayer coatings, using identical deposition conditions as for *M*Co-DLC3. However, for this coating, the power to both pure graphite targets was turned off and only the ‘C + Co’ target was active. The Co amount of 38 at.% was measured by EDS, and, hence, this coating was named *M*Co-DLC38. This is the amount of Co present in the geometrically central part of Co-DLC layers of *M*Co-DLC3; this concentration is the value when, during the deposition of *M*Co-DLC3, the substrates were exactly in front of the ‘C + Co’ target during rotation.

Considering this value and maintaining proportionality between EDS measurements shown in Table 2, it was possible to estimate the maximum Co concentration for the *M*Co-DLC1 and *M*Co-DLC2 coatings (18 and 30 at.%, respectively). Therefore, the geometrical central parts of Co-DLC layers in multilayer coatings had 18, 30, and 38 at.% Co for *M*Co-DLC1, *M*Co-DLC2, and *M*Co-DLC3, respectively.

The XRD diffraction analysis (θ–2θ mode/Bragg–Brentano geometry, and grazing incidence) of the coatings did not show any crystalline peaks from C, Co, or Co-C carbides indicating their amorphous nature. These results are not shown pictorially.

**Figure 6:**
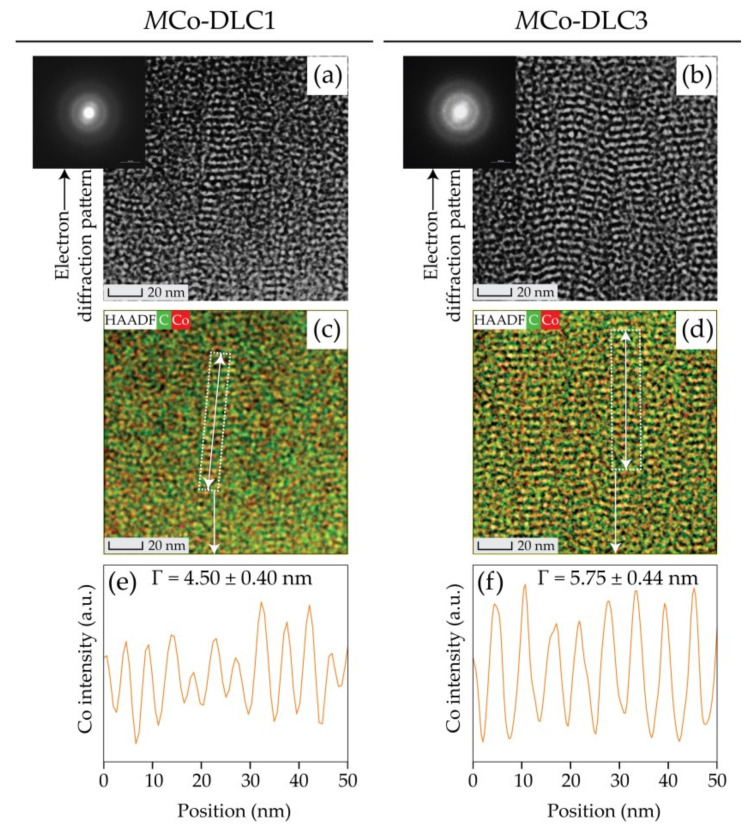
The results of TEM for multilayer *M*Co-DLC1 and *M*Co-DLC3 coatings: (**a**,**b**) high-resolution HAADF TEM images and electron diffraction patterns (EDPs) obtained by 4D-STEM, (**c**,**d**) TEM-EDS elemental maps for C and Co, and (**e**,**f**) Co intensity along the EDS line scan as shown by a white line and rectangle in (**c**,**d**).

The internal and multilayer structure of *M*Co-DLC1 and *M*Co-DLC3 (coatings with minimum and maximum dopant concentration) was analysed by TEM. The results obtained are shown in Figure 6. In this figure, the symbol ‘Γ’ represents the period between two consecutive layers of DLC and Co-DLC in the coatings. The detailed TEM analysis provides insight into the structure and the distribution of the Co dopant in the coatings.

The multilayer structure of *M*Co-DLC1 and *M*Co-DLC3 coatings is visible in the high-resolution TEM images shown in Figure 6a,b. The multilayer structure is more prominent in the *M*Co-DLC3 coatings compared to *M*Co-DLC1, due to the higher period between the undoped and doped layers, resulting from the higher power applied to the ‘C + Co’ target. To confirm the existence of the proposed multilayers, EDS was performed during TEM analysis for the images shown in Figure 6a,b. The superimposed elemental maps of C and Co along with HAADF images are presented in Figure 6c,d. These maps confirm the multilayer structure based on the distribution of elements.

To further analyse the characteristics of this multilayer structure, an EDS line scan was performed along the lines indicated by white rectangles in Figure 6c,d. The results of this line scan are shown in Figure 6e,f as distance versus Co intensity, where the crests represent Co-DLC layers, and the troughs represent DLC layers. The period between these layers was quantified by measuring the distance difference between the crests. The measured periods were 4.50 ± 0.40 nm for *M*Co-DLC1 and 5.75 ± 0.44 nm for *M*Co-DLC3. These measured period values are in tune with the calculated ones (4.49 and 5.34 nm for the *M*Co-DLC1, and *M*Co-DLC3, respectively). This comparison validates the methodology used to create these multilayer coatings.

During the TEM analysis, pixel-by-pixel electron diffraction patterns were obtained for the high-resolution images. The representative electron diffraction patterns for *M*Co-DLC1 and *M*Co-DLC3 are shown in Figure 6a,b. These diffraction patterns showed only halo ring formations, confirming that the coatings were amorphous.

**Figure 7:**
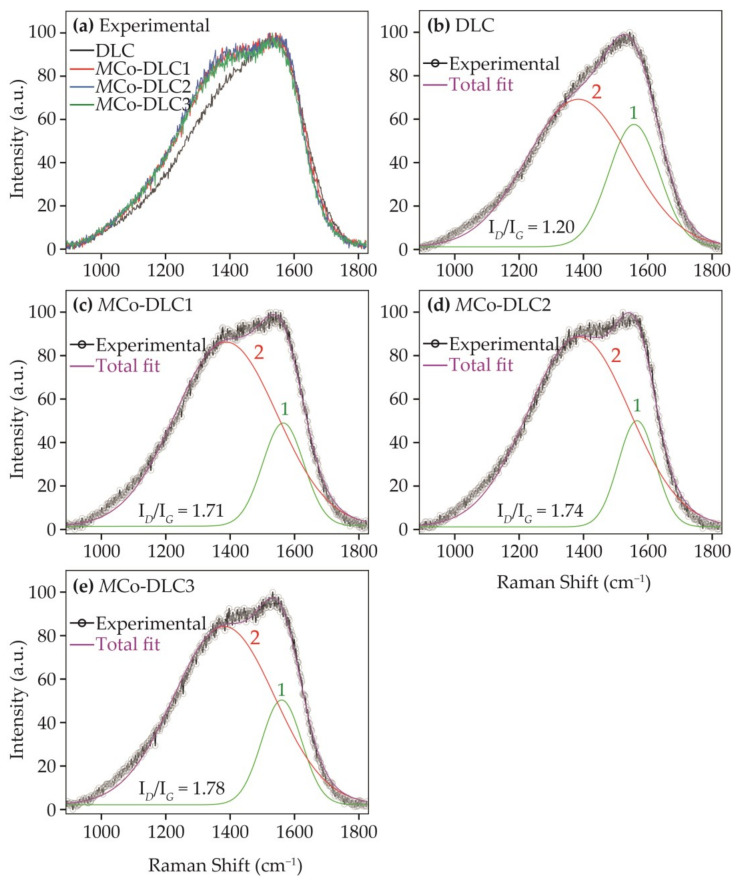
The results of Raman spectroscopic analysis: (**a**) normalised data without peak fitting for all coatings. Peak-fitted results of (**b**) DLC, (**c**) *M*Co-DLC1, (**d**) *M*Co-DLC2, and (**e**) *M*Co-DLC3 coatings. In these figures, ‘1’ represents the *G* peak and ‘2’ represents the *D* peak.

Raman spectroscopic analysis was performed to evaluate the effect of the dopant on the extent of graphitisation in the coatings. The resulting non-peak fitted and normalised spectra are shown in Figure 7a for DLC, *M*Co-DLC1, *M*Co-DLC2, and *M*Co-DLC3. The peak-fitted results for all these coatings are presented in Figure 7b–e.

All the normalised spectra showed a broad band consisting of two superimposed peaks in the Raman shift range of 890–1830 cm^−1^, as shown in Figure 7a. These normalised curves for all coatings were fitted with two peaks (peaks of *D* and *G* bands) using a Gaussian function and are shown in Figure 7b–e. According to the literature [45], the *D* peak in DLC coatings is ascribed to the breathing mode of sp^2^-bonded atoms in the rings; this peak is situated around 1300–1450 cm^−1^. The *G* peak, which appears due to bond stretching in the pairs of sp^2^-bonded atoms in the rings and chains, is situated around 1530–1580 cm^−1^ [45,46]. After peak fitting, the I*_D_*/I*_G_* ratios were calculated. In a given system of DLC coatings, an increase in the I*_D_*/I*_G_* ratio is attributed to a higher extent of graphitisation in the coating [45], and vice versa.

The undoped DLC coating showed an I*_D_*/I*_G_* ratio of 1.20, whereas *M*Co-DLC1, *M*Co-DLC2, and *M*Co-DLC3 all showed an I*_D_*/I*_G_* ratios of ≈1.7. Here, it can be inferred that the addition of Co in DLC to form Co-DLC coatings increased graphitisation in our studied system, but the I*_D_*/I*_G_* ratios remained consistent once the Co was doped in the DLC for all the Co-doped coatings irrespective of the doping concentration of the dopant. This observation is in agreement with the published literature where authors have proven that the inclusion of Co does not change the extent of graphitisation in the DLC coatings with its increasing or decreasing amount [18,30,31,32], as was also discussed in Section 1.

### 3.2. Mechanical Properties

The mechanical properties of undoped and multilayer Co-DLC coatings, along with the values of residual stresses and I*_D_*/I*_G_* ratios are shown in Table 3.

The undoped DLC coating had a hardness value of 16.2 GPa and a reduced modulus of 184.8 GPa. The addition of Co induced a decrease in hardness and the reduced modulus of Co-doped multilayer coatings. To form the *M*Co-DLC1 by adding 4.1 at.% Co into the DLC, the hardness value was reduced to 13.4 GPa, and a reduction in the reduced modulus occurred with a value of 158.6 GPa. With the further addition of 6.9 and 8.7 at.% Co in the coatings to fabricate the *M*Co-DLC2 and *M*Co-DLC3, the hardness further reduced to 12.4 and 11.1 GPa, respectively. Additionally, the reduced modulus decreased to 155.6 and 149.2 GPa, respectively. When comparing the hardness and reduced modulus of undoped DLC with the Co-doped multilayer coatings, the decrease in hardness was 18.9%, 26.5%, and 37.3% for *M*Co-DLC1, *M*Co-DLC2, and *M*Co-DLC3, respectively. Similarly, the reduced modulus of these coatings decreased by 15.2%, 17.1%, and 21.3%, respectively, compared to undoped DLC. The H/E* and H^3^/E*^2^ values (Table 3) are representative of the elastic/plastic response of the coatings [33]. Both of these parameters followed the identical trend as the hardness and the reduced modulus. The W_e_ of the *M*Co-DLC1 showed a significant improvement as compared to all the other coatings.

**Figure 8:**
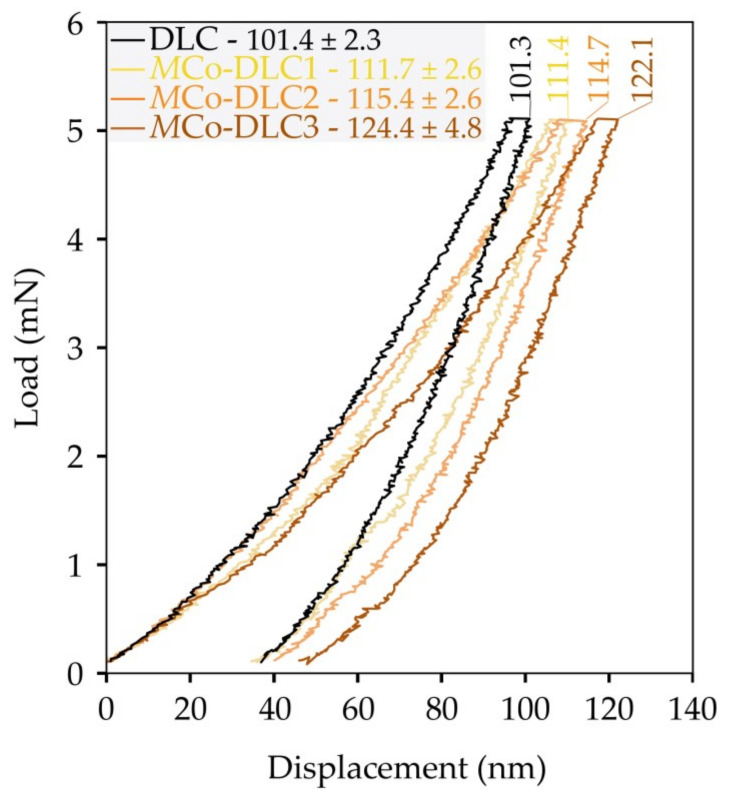
The load vs. displacement curves for all the coatings. The values marked on the curves correspond to the individual plots shown. The values displayed alongside the coating names represent the average values with standard deviation.

In Figure 8, the load vs. displacement curves for all the coatings studied in this work are presented. Each coating is represented by a single plot, with its respective indentation depth marked above the curve. The average indentation depth values for all coatings are indicated in the legend box alongside their respective coating names. It can be observed that the smallest indentation depth was recorded for DLC due to its higher hardness. As Co dopant was introduced into the coatings, the indentation depth increased progressively, with the coating containing the highest cobalt concentration exhibiting the greatest indentation depth and the lowest hardness, and vice versa. In all cases, the indentation depth remained below 10% of the total coating thickness.

**Figure 9:**
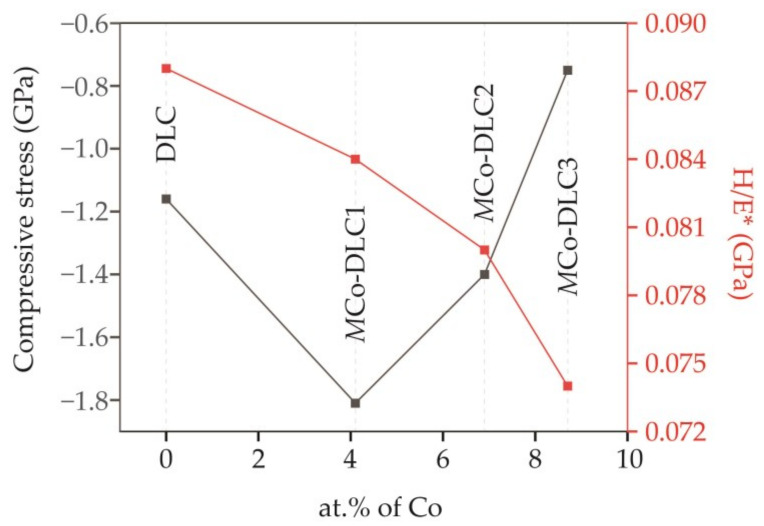
Residual compressive stress and H/E* for DLC and multilayer *M*Co-DLC coatings as a function of Co concentration.

The compressive stresses in the DLC coatings originate from several factors, including the mismatch of thermal expansion between the substrate and coating, the concentration and atomic size of the dopant, and the thickness of the coating [37]. All the coatings studied in this work showed residual stresses that were compressive in nature. The undoped DLC showed a compressive stress of −1.16 GPa. With the addition of 4.1 at.% of Co, the residual stresses increased noticeably initially for *M*Co-DLC1 (−1.81 GPa) and then started to decrease for *M*Co-DLC2 and *M*Co-DLC3 with values of −1.40 and −0.75 GPa, respectively. Initially, the stress increased due to the inclusion of larger Co atoms in the matrix of smaller C atoms, generating a stress field in the DLC. The covalent atomic radius of C is 170 pm, whereas it is 192 pm for Co, a difference of 12.2%, which is significant. With a further increase to 6.9 and 8.7 at.% of Co, the stresses started to decrease due to the accumulation of metallic Co in the layers of Co-DLC in the multilayer structure. The trend of residual stresses along with the H/E* trend is shown in Figure 9 as a function of Co concentration.

From the data presented in Table 3, it can be inferred that the mechanical properties of the coatings developed in this work depend on the concentration of the dopant and the residual stresses. However, contrary to most studies which describe the mechanical properties based on I*_D_*/I*_G_* ratios, this relationship is not obvious in the case of Co dopant while considering Co-DLC coatings. The I*_D_*/I*_G_* ratios were consistent across the Co-doped DLC coatings regardless of the concentration of the Co dopant, indicating that the extent of graphitisation did not vary significantly with different Co concentrations, the maximum difference in I*_D_*/I*_G_* within Co-DLC coatings was 0.07 equivalent 4%. This suggests that the mechanical properties are more substantially influenced by the dopant concentration and the residual stresses rather than the degree of graphitisation indicated by the I*_D_*/I*_G_* ratio.

**Figure 10:**
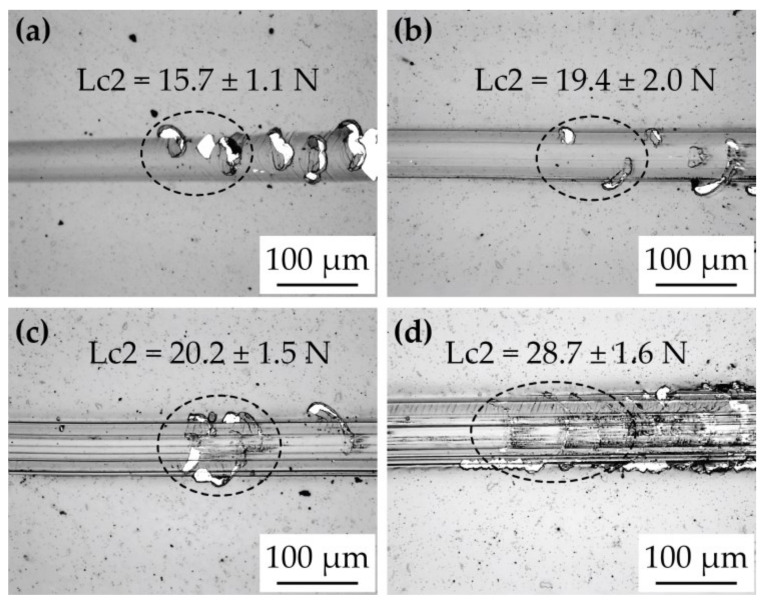
The results of the scratch adhesion test and values of adhesive failure (Lc2) for (**a**) DLC, (**b**) *M*Co-DLC1, (**c**) *M*Co-DLC2, and (**d**) *M*Co-DLC3.

The optical images of the morphology of scratches obtained by the travelling optical microscope are shown in Figure 10. The numerical values of the adhesive failure—Lc2/practical adhesion—are mentioned in their respective figures. These values are an average of five scratch passes for each coating, and the standard deviation is also provided with the Lc2 values.

The undoped DLC coating showed Lc2 adhesive failure at 15.7 ± 1.1 N. All the Co-doped coatings exhibited higher adhesion compared to the undoped DLC, with values of 19.4 ± 2.0, 20.2 ± 1.5, and 28.7 ± 1.6 N for *M*Co-DLC1, *M*Co-DLC2, and *M*Co-DLC3, respectively. The Lc2 values for *M*Co-DLC1 and *M*Co-DLC2 differed by a small amount, reflecting the minimal difference in their compressive residual stresses. A significant improvement in adhesion was observed for *M*Co-DLC3, which could be attributed to the substantial reduction in internal stresses and the lowest hardness among the coatings.

### 3.3. Tribological Analysis

All the coatings produced in this work, including DLC, *M*Co-DLC1, *M*Co-DLC2, and *M*Co-DLC3, were tested tribologically. An uncoated but polished AISI M2 steel substrate was used as a reference. The tests were performed under oil-lubricated conditions using pure base oil (BO) and six different oil formulations (three formulations of BO-X-178 and three formulations of BO-X-OAP).

**Figure 11:**
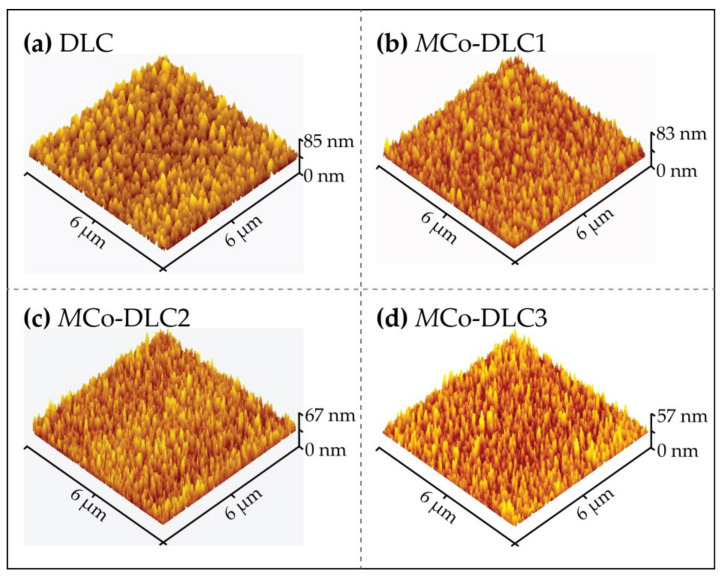
AFM topographical 3D images for (**a**) DLC, (**b**) *M*Co-DLC1, (**c**) *M*Co-DLC2, and (**d**) *M*Co-DLC3.

The surface roughness of the polished steel sample and all the coatings were measured using WLI and AFM to calculate the tribological test parameters. AFM topographical images for all the coatings are shown in Figure 11.

For all the samples, R_q_ values between 10.3 and 14.4 nm were measured. Identical roughness values were achieved from both techniques when the normalised scan areas were considered. The measured roughness (R_q_) values for DLC, *M*Co-DLC1, *M*Co-DLC2, and *M*Co-DLC3 were 10.3, 14.4, 13.1, and 11.0 nm, respectively. The roughness values exhibited a direct one-to-one correlation with the granular sizes calculated, as shown in Figure 4 and discussed in the accompanying text.

**Figure 12:**
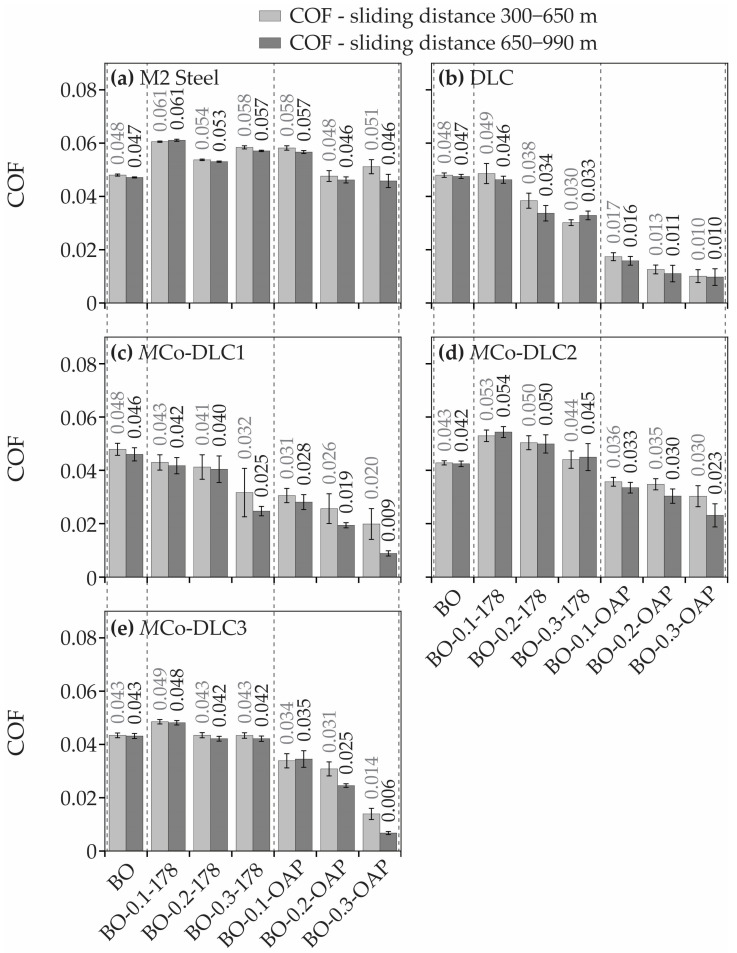
COF values obtained for (**a**) uncoated and polished AISI M2 steel, (**b**) DLC, (**c**) *M*Co-DLC1, (**d**) *M*Co-DLC2, and (**e**) *M*Co-DLC3 when tested with base oil and all the oil formulations.

The measured COF values for polished steel, DLC, and multilayer *M*Co-DLC coatings when tested with base oil and all the oil formulations are shown in Figure 12. Every test was repeated three times. The results shown in Figure 12 are after 300 m of sliding distance, after reaching the steady-state friction conditions. The COF values are shown for two sliding distance intervals after reaching the steady state conditions, one set of values are the COF values between 300 and 650 m of sliding distance and other ones are for sliding distance 650–990 m.

**Figure 13:**
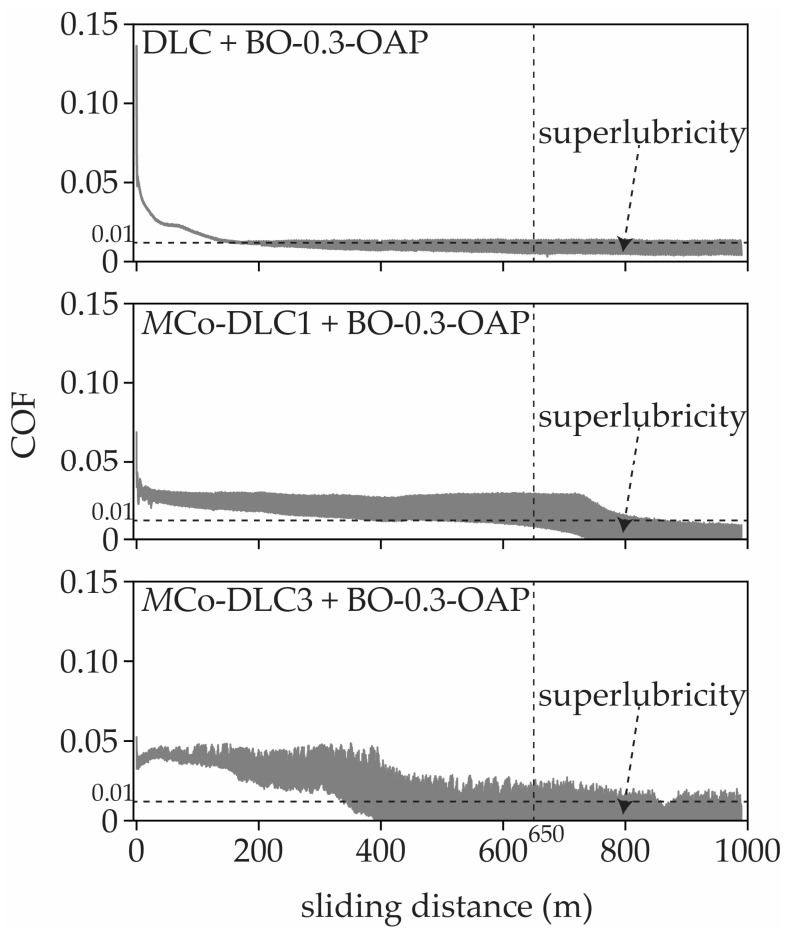
The COF vs. sliding distance curves for the combination of coatings and oil formulations where superlubricity was observed after 650 m of sliding distance.

From the presented values, it can be seen that additives in the base oil did not influence COF (0.047–0.061) for polished and uncoated steel disc samples. When undoped DLC and multilayer *M*Co-DLC coatings are considered, it is evident that BO-X-OAP oil formulations reduced the COF values significantly as compared to that of BO and BO-X-178 oil formulations. Within the BO-X-178 oil formulations, the COF decreased progressively with each composition, but the reduction was not as substantial as with BO-X-OAP oil formulations. The lowest COF values for COF for all the coatings were observed when tested with BO-0.3-OAP, having lowest values of 0.010, 0.009, 0.023, and 0.006 for DLC, *M*Co-DLC1, *M*Co-DLC2, and *M*Co-DLC3, respectively. All the test results were in two intervals (300–650 m and 650–990 m) after reaching steady-state conditions, and the friction curves were smooth and consistent. However, for DLC, *M*Co-DLC1, and *M*Co-DLC3 tested with BO-0.3-OAP, a sudden decrease in COF was observed after almost 600 m of sliding distance, with COF values dropping and approaching the values 0.006 showing superlubricity. The COF values seemingly dropped below 0.006, but our load cell was not designed to measure the COF values below 0.006. The values kept on decreasing until the test finished, indicating that more future work is required to fully understand the friction behaviour of these three coatings by using a precise enough load cell with more resolution as compared to that used in this study. In the coatings, where superlubricity was observed and COF values almost touched zero values are shown in Figure 13.

**Figure 14:**
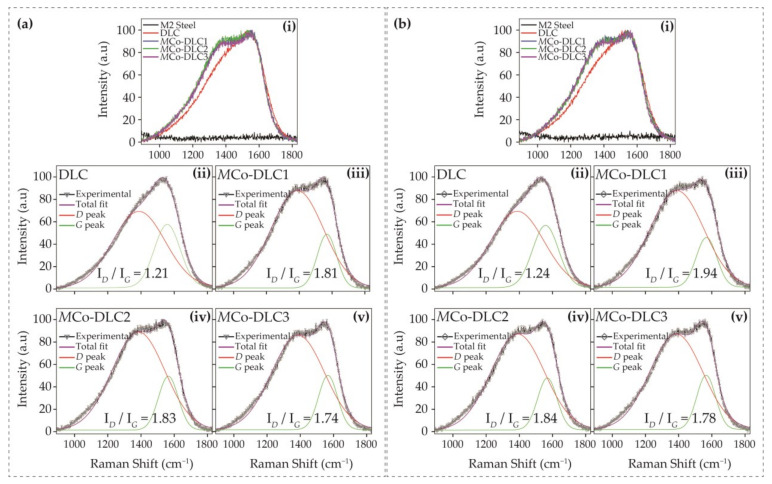
The results of Raman spectroscopic analysis performed on the wear tracks on post tribological tested surfaces of the discs. (**a**) For all the disc specimens tested with BO-0.3-178 oil formulation: (**ai**) normalised and non-peak fitted curves for all the disc specimens, (**aii**–**av**) normalised and peak fitted curves along with I*_D_*/I*_G_* ratios for DLC, *M*Co-DLC1, *M*Co-DLC2, and *M*Co-DLC3, respectively and (**b**) for all the disc specimens tested with BO-0.3-OAP oil formulation: (**bi**) normalised and non-peak fitted curves for all the disc specimens, (**bii**–**bv**) normalised and peak fitted curves along with I*_D_*/I*_G_* ratios for DLC, *M*Co-DLC1, *M*Co-DLC2, and *M*Co-DLC3, respectively.

To further explain the behaviour of COF in relation to rubbing-induced graphitisation and the interaction with phosphorus-containing additives, Raman spectroscopic analysis was conducted on the tribologically tested disc samples. The results are presented in Figure 14.

In Figure 14a, the results are shown for the discs tested with the BO-0.3-178 oil formulation. Figure 14(ai) presents the non-peak-fitted combined results, while Figure 14(aii–av) displays the peak-fitted results for the DLC, *M*Co-DLC1, *M*Co-DLC2, and *M*Co-DLC3 coatings when tested with BO-0.3-178. The I*_D_*/I*_G_* ratios are also marked in the figures. Similarly, Figure 14b shows the results for the discs tested with the BO-0.3-OAP oil formulation for all disc specimens. In Figure 14(bi), the non-peak fitted and normalised results are shown in a combined manner, whereas Figure 14(bii–bv) presents the peak-fitted results along with the I*_D_*/I*_G_* ratios.

It was observed that uncoated M2 steel discs did not show any Raman signal from carbon, indicating that the base oil and additives used did not induce the deposition of a rubbing or friction-induced carbon layer on the discs. However, for the DLC and *M*Co-DLC coated discs, the I*_D_*/I*_G_* ratios generally increased as a result of the rubbing between the discs and counter bodies during the tribological tests. This increase in I*_D_*/I*_G_* ratios suggests enhanced graphitisation of the coatings. By comparing Figure 14a and Figure 14b side by side, it can be seen that the I*_D_*/I*_G_* ratios increased substantially when the BO-0.3-OAP oil formulation was used, as compared to the BO-0.3-178 formulation. Notably, for the coatings that exhibited superlubricity (Figure 13), the I*_D_*/I*_G_* ratios increased significantly. For *M*Co-DLC1, the ratio increased from 1.71 to 1.94 (a 12.6% increase), whereas for *M*Co-DLC3, the ratio remained constant, similar to the as-deposited coating. The lower COF value for *M*Co-DLC1 can be attributed to the increased graphitic nature. For all the coatings, the mechanical behaviour and tribological properties were influenced by a combination of factors, including mechanical properties, the extent of graphitisation, dopant concentration, the period between layers, and residual stresses. The decrease in COF for *M*Co-DLC3 can be attributed to the thicker Co-DLC layers, minimal residual stresses, the lowest hardness among all coatings, and, importantly, the Raman analysis of the counterbodies discussed subsequently. No indication of phosphorus-containing species was observed in the Raman analysis, likely due to the very low concentration of phosphorus in the oil formulations (a maximum of only 0.3 wt.%).

A direct one-to-one correlation between the surface roughness (R_q_) and granular sizes of the coatings with the COF could not be established, as COF is influenced by multiple factors beyond surface roughness, as discussed in the preceding paragraph. However, in general, lower COF values were observed for coatings with lower surface roughness and smaller granular sizes.

**Figure 15:**
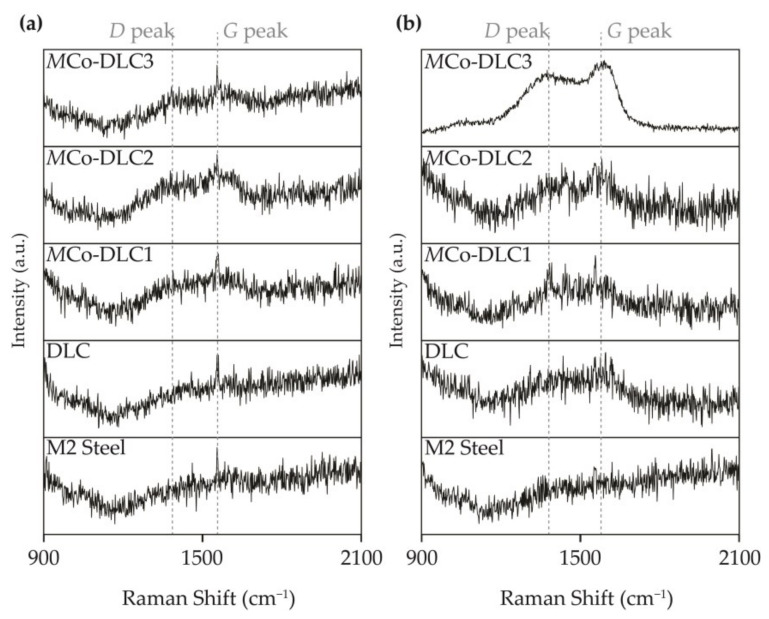
The results of the Raman analysis performed on the wear scars present on the counterbodies after tribological tests, (**a**) when tested with BO-0.3-178 oil formulations and all the discs specimens, and (**b**) when tested with BO-0.3-OAP and all the disc specimens.

To further clarify COF behaviour, Raman analysis was also performed on the wear scars on the steel counterbodies after the tribological tests. The results of this analysis are shown in Figure 15.

The curves shown in Figure 15 could not be peak-fitted to assess the nature of the species present due to their low intensity and higher noise levels. However, they provided crucial qualitative information. From Figure 15a,b, it can be observed that no carbon was present on the wear scars when the counterbodies were rubbing against the polished M2 steel disc, further confirming the Raman wear track analysis of the discs. In contrast, a carbon layer was consistently present on the wear scars of the counterbody balls when tested with DLC-based coatings, as indicated by the clear presence of the *D* and *G* bands in the Raman spectra. This is the carbon transferred from the discs to the balls during tribological tests. Notably, when the counterbody ball which was rubbing against *M*Co-DLC3 + BO-0.3-OAP, a clear signal of highly graphitic carbon was detected, which may have contributed to the exceptionally low COF value of 0.006 for this coating.

**Figure 16:**
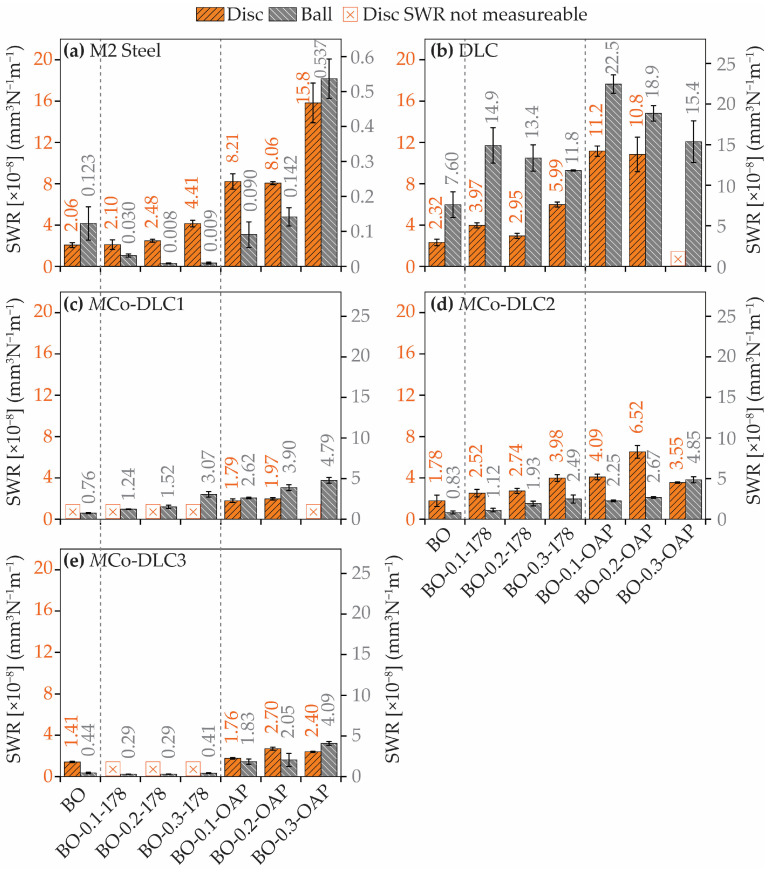
SWR values obtained for (**a**) uncoated AISI M2 steel, (**b**) DLC, (**c**) *M*Co-DLC1, (**d**) *M*Co-DLC2, and (**e**) *M*Co-DLC3 when tested with BO and all the oil formulations. The *Y*-axis values of the ball wear for M2 steel are not the same as those in the other plots (consistent for all other plots). The cross mark corresponds to disc samples where the wear was so small that it was not measurable.

The SWR values for the tribologically tested discs and balls are presented in Figure 16. The orange columns represent the SWR of the discs, whereas the dark grey columns show the SWR of the balls. In some tests, the SWR was not measurable due to very small wear volumes; these instances are indicated by a cross mark in Figure 16.

For the M2 steel disc samples, the SWR values varied between 2.06 and 15.8 × 10^−8^ mm^3^·N^−1^·m^−1^, while for the counter body balls, the SWR values ranged from 0.008 to 0.537 × 10^−8^ mm^3^·N^−1^·m^−1^. As seen in Figure 16, neither the BO nor the oil formulations effectively reduced the SWR of the discs; in fact, the wear increased when BO-X-OAP oil formulations were used. Conversely, a reduction in SWR for the balls was noted when BO-X-178 oil formulations were utilised.

For the undoped DLC, the SWR on the disc was reduced compared to bare steel, but this led to a significant increase in the SWR of the balls. All the multilayer *M*Co-DLC coatings demonstrated a reduction in SWR compared to both bare steel and undoped DLC. Notably, a significant reduction in SWR was observed with the BO-X-178 oil formulations, to the extent that some SWR values were too low to measure.

Similarly to COF, a generalised trend between the surface roughness of the coatings and SWR was not observed. To isolate the effect of surface roughness and minimise the influence of additives in the lubricating oil formulations, only tests conducted with BO were considered for this argument. It was observed that the highest SWR of the counterbody ball occurred with the DLC coating, likely due to its high hardness. Among the multilayer doped coatings, the SWR of the counterbody balls initially became unmeasurable, then increased, and subsequently decreased. A similar trend was observed for the SWR of the discs.

**Figure 17:**
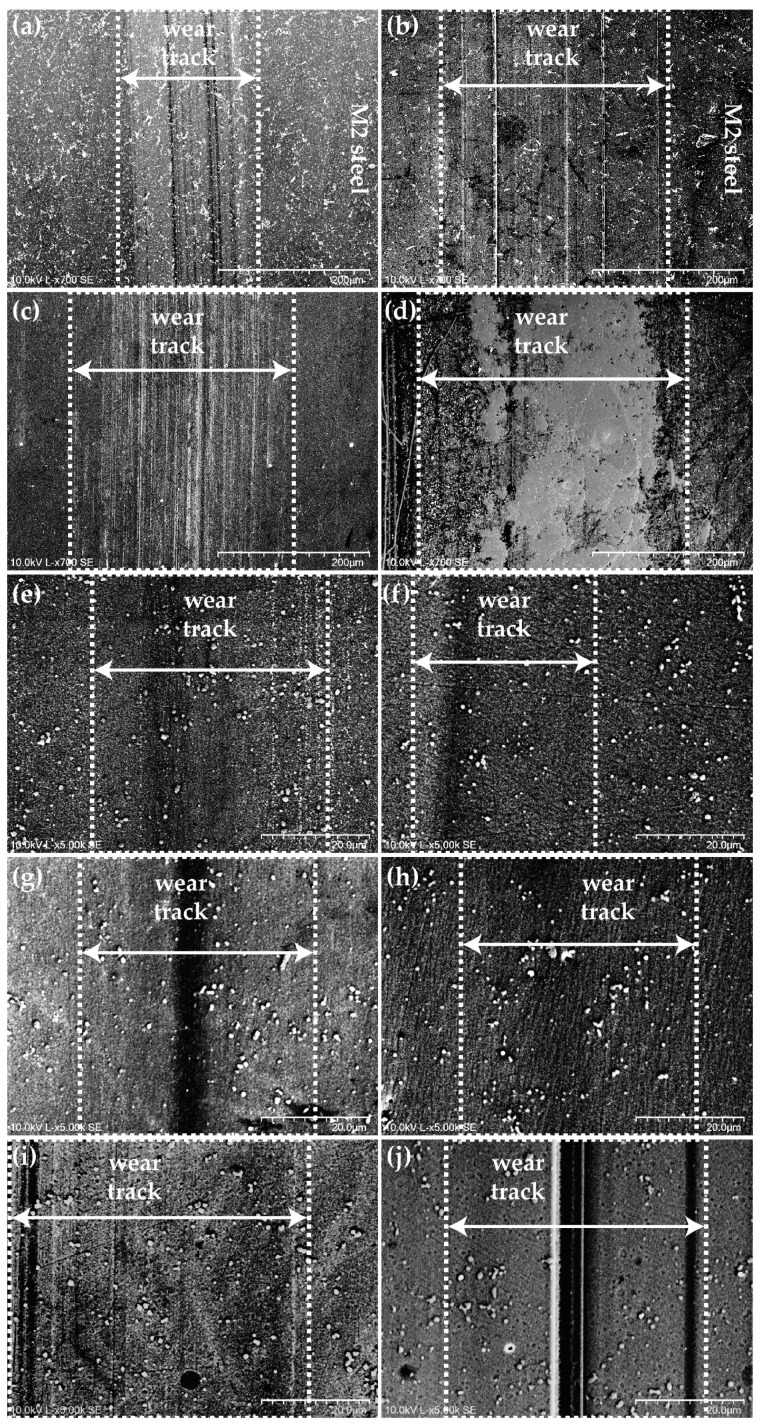
SEM micrographs of the wear tracks present on the discs after completion of the tribological tests. (**a**) M2 steel + BO-0.3-178, (**b**) M2 steel + BO-0.3-OAP, (**c**) DLC + BO-0.3-178, (**d**) DLC + BO-0.3-OAP, (**e**) *M*Co-DLC1 + BO-0.3-178, (**f**) *M*Co-DLC1 + BO-0.3-OAP, (**g**) *M*Co-DLC2 + BO-0.3-178, (**h**) *M*Co-DLC2 + BO-0.3-OAP, (**i**) *M*Co-DLC3 + BO-0.3-178, and (**j**) *M*Co-DLC3 + BO-0.3-OAP.

To further understand tribological aspects such as SWR and COF, as well as the presence of possible transfer layers or tribofilms, SEM analysis was performed on the wear tracks of the discs, coupled with EDS measurements. The results from SEM imaging of the wear tracks on the discs are presented in Figure 17.

In Figure 17, post-tribological test wear tracks on the discs are shown for all the disc samples, for the micrographs obtained by SEM. The wear tracks are aligned in the sliding direction, with wear and debris visible for all samples. The wear observed on the tracks visually matches the quantitative values presented in Figure 16. The wear was highest on the uncoated M2 steel discs and the DLC coatings, making the wear tracks more prominent in these cases. For the *M*Co-DLC coatings, wear was measurable when tested with BO-X-OAP oil formulations, and the wear is visible in this case. However, wear was not measurable when the coatings were tested with BO-X-178 oil formulations. In the SEM micrographs, shallow wear tracks can be seen, which explains why wear was not measurable using WLI for the coatings tested with oil formulations of BO-X-178.

**Figure 18:**
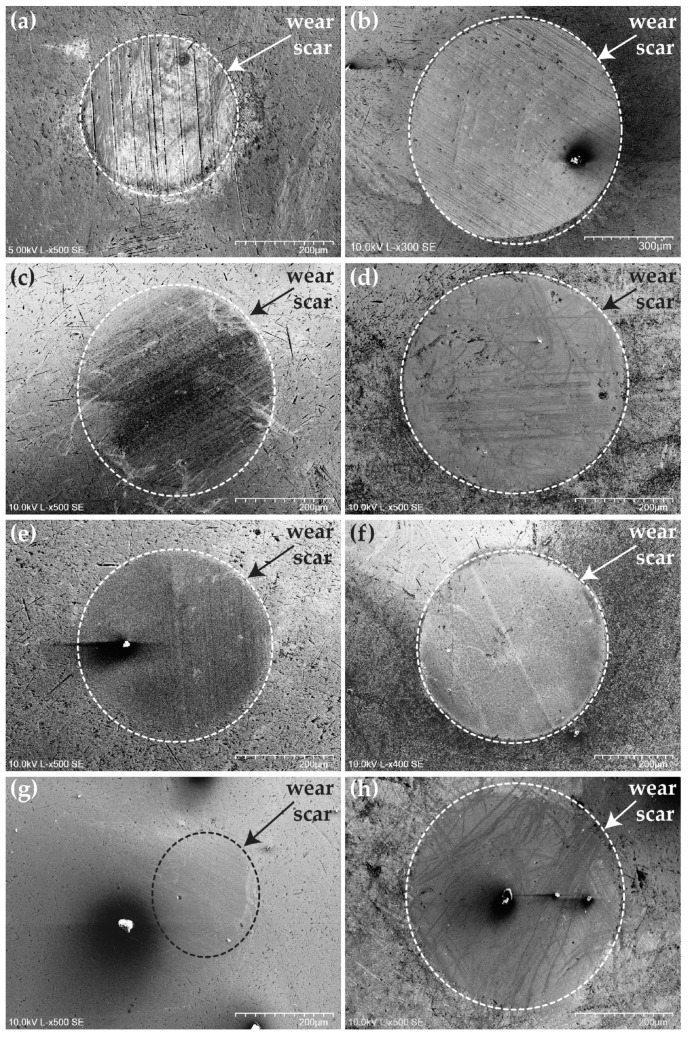
SEM micrographs of the wear scars present on the counterbody balls after completion of the tribological tests. (**a**) M2 steel + BO-0.3-OAP, (**b**) DLC + BO-0.3-OAP, (**c**) *M*Co-DLC1 + BO-0.3-178, (**d**) *M*Co-DLC1 + BO-0.3-OAP, (**e**) *M*Co-DLC2 + BO-0.3-178, (**f**) *M*Co-DLC2 + BO-0.3-OAP, (**g**) *M*Co-DLC3 + BO-0.3-178, and (**h**) *M*Co-DLC3 + BO-0.3-OAP. Magnification is not the same for the images.

In Figure 18, SEM micrographs of the wear scars on the counterbody balls after the tribological tests are shown for the balls tested with all oil formulations and disc samples.

The wear debris and scratches resulting from wear are apparent in these images. A discontinuous carbon transfer film can also be observed on the wear scars, as confirmed by the Raman analysis in Figure 15. For the counterbody balls, the highest wear was observed for those tested against the DLC coatings, resulting in the largest and most prominent wear scar in Figure 18b. The lowest wear was calculated for the balls tested against the uncoated M2 steel, which is in full agreement with the SEM micrographs, showing the smallest wear scar diameter in Figure 18a. The wear scar diameters in Figure 18 directly correspond to the SWR values presented in Figure 16.

**Figure 19:**
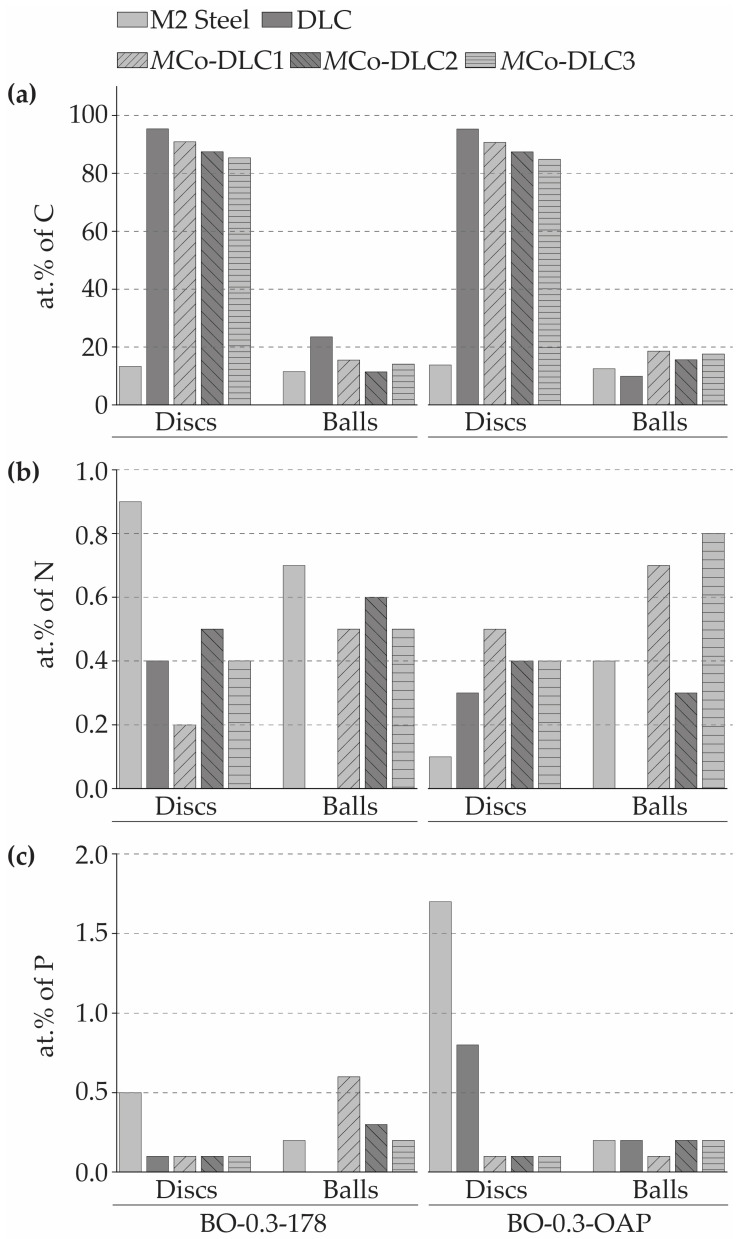
EDS of worn-out post tribological test surfaces. (**a**) carbon, (**b**) nitrogen, and (**c**) phosphorus.

To further reason the decrease in COF by BO-X-OAP oil formulations and the decrease in SWR by the BO-X-178 oil formulations, EDS analysis was performed on the post-tribological wear tracks and wear scars. Although EDS is not an ideal method for detecting lightweight elements, when combined with Raman spectroscopy and SEM imaging, it can provide valuable comparative insights into tribological phenomena. The elemental species carbon, nitrogen, and phosphorus were measured on the discs and balls when tested with BO-0.3-178 and BO-0.3-OAP (Figure 19).

The amount of carbon present on the discs did not change significantly before and after the tribological tests, but the amount of carbon transferred from the disc to the balls was substantial (the carbon content in the AISI 52100 steel is about 3.5 at.%). In general, the amount of transferred carbon was higher when BO-X-OAP oil formulations were used as compared to BO-X-178 formulations. This suggests that BO-X-OAP oil formulations triggered some form of graphitisation in the coating’s carbon, which may have contributed to reducing friction. This observation further supports the Raman analysis results of the counterbody balls.

The amount of phosphorus detected on the balls and discs was higher when tested with BO-X-OAP oil formulations, as this formulation contained nearly double the phosphorus compared to BO-X-178. This higher phosphorus content in BO-X-OAP helped reduce the COF to the extent that superlubricity was achievable, likely by forming phosphorus-containing species on both the balls and discs. Regarding nitrogen, a significant amount was detected on the balls and discs when BO-X-178 oil formulations were used, as Duraphos^®^ 178 additive contains an amine group in its structure, whereas Duraphos^®^ OAP does not. According to the literature, the presence of an amine group helps stabilise tribologically induced species and enhances their durability, which contributes to the lower SWR values.

## 4. Conclusions

- Multilayer Co-DLC/DLC coatings with cobalt concentrations of 4.1, 6.9, and 8.7% were successfully developed using non-reactive direct current magnetron sputtering (DCMS).
- The multilayer structure was achieved by controlling the angular speed of the substrate holder during deposition, with 1 rpm used for the deposition of multilayer Co-doped DLC coatings.
- All coatings exhibited granular, cauliflower-like surface morphology and columnar cross-sectional morphology. The ‘carbon + chromium’ gradient interlayer had an average thickness of 0.33 µm, while the coatings measured an average thickness of 1.07 µm.
- X-ray diffraction and electron diffraction analyses confirmed that all coatings were amorphous.
- The multilayer microstructure was first calculated based on the deposition rates and later confirmed through transmission electron microscopy (TEM) coupled with energy dispersive spectroscopy (EDS). The calculated and measured values were in agreement, validating the methodology.
- The cobalt doping did not significantly affect the I*_D_*/I*_G_* ratios in any of the Co-doped DLC coatings.
- The addition of cobalt reduced hardness and the reduced modulus, but increased toughness (W_e_).
- All coatings exhibited compressive residual stresses.
- Adhesion was significantly improved by cobalt doping, with the Co-DLC coating containing 8.7% cobalt showing the greatest improvement, 59% higher than undoped DLC.
- Co-DLC coatings tested with BO-X-OAP oil formulations (PAO4 + C_8_H_19_PO_4_) achieved superlubricity, with COF values reaching as low as 0.006. The I*_D_*/I*_G_* ratios increased after the tribological tests, more significantly with ‘PAO4 + C_8_H_19_PO_4_’ than with BO-X-178 oil formulations (PAO4 + C_19_H_42_NPO_4_—C_29_H_63_NPO_4_).
- A reduction in wear was observed for the coatings when tested with BO-X-178 oil formulations (PAO4 + C_19_H_42_NPO_4_—C_29_H_63_NPO_4_).

## Figures and Tables

**Table 1:** Nomenclature of the deposited coatings and main deposition parameters.

Coatings	DLC	*M*Co-DLC1	*M*Co-DLC2	*M*Co-DLC3
Power at C + Co target (W)	0	160	305	450
Power at C targets (W)	1750	1750	1750	1750
Deposition time (sec)	15,480	14,340	13,260	12,240
Rotational speed of substrate holder (rpm)	12	1	1	1

**Table 2:** Chemical composition of the coatings assessed by EDS.

Coating	SEM-EDS (at.%)			
	C	Co	Ar	O
DLC	96.2	0.0	3.5	0.3
*M*Co-DLC1	91.7	4.1	3.8	0.4
*M*Co-DLC2	88.7	6.9	3.6	0.8
*M*Co-DLC3	86.0	8.7	3.4	1.9

**Table 3:** Values of mechanical properties (H, E*, W_e_, H/E*, and H^3^/E*^2^), residual stresses, and I*_D_*/I*_G_* ratios.

Coating	Co (at.%)	H (GPa)	E* (GPa)	W_e_ (%)	H/E*	H^3^/E*^2^ (GPa)	Residual Stresses (GPa)	I*_D_*/I*_G_*
DLC	0.0	16.2 ± 0.6	184.8 ± 5.7	63.7	0.088	0.124	−1.16 ± 0.06	1.20
*M*Co-DLC1	4.1	13.4 ± 1.1	158.6 ± 6.4	66.0	0.084	0.096	−1.81 ± 0.09	1.71
*M*Co-DLC2	6.9	12.4 ± 0.9	155.6 ± 6.3	63.6	0.080	0.079	−1.40 ± 0.20	1.74
*M*Co-DLC3	8.7	11.1 ± 0.8	149.2 ± 4.9	59.2	0.074	0.061	−0.75 ± 0.09	1.78

## Data Availability

The original contributions presented in this study are included in the article. Further inquiries can be directed to the corresponding authors.
